# Multi-Omics for the Understanding of Brain Diseases

**DOI:** 10.3390/life11111202

**Published:** 2021-11-07

**Authors:** Chiara Villa, Jong Hyuk Yoon

**Affiliations:** 1 School of Medicine and Surgery, University of Milano-Bicocca, 20900 Monza, Italy; 2 Neurodegenerative Diseases Research Group, Korea Brain Research Institute, Daegu 41062, Korea

Brain diseases, including both neurodegenerative diseases and mental disorders, represent the third largest healthcare problem in developed countries, after cardiovascular disorders and cancer [1]. The majority of human brain diseases have a multifactorial etiology and are characterized by different molecular alterations that act in synergy during the disease development [2]. In the last decade, the advancement of omics technologies, such as genomics, transcriptomics, proteomics, epigenomics, metabolomics, miRNomics, and lipidomics, offers a great contribution to the identification of novel molecular pathways, and to understand pathophysiological alterations underlying brain diseases. However, molecular profiling, which makes it possible to deepen the understanding of these disorders, has shown some limitations. Therefore, the analysis and integration of data derived from massively parallel technologies will allow the simultaneous identification of molecular alterations at different levels (transcript, gene, microRNA, protein, lipid, cellular metabolic processes), incorporating the available information, and thus contributing to providing novel insights into the mechanisms underlying human brain diseases [3].

This Special Issue gathers four reviews and six original research articles highlighting the potentialities of omics approaches in different brain diseases. Some authors reviewed the applications and impact of microfluidics technology on research in Alzheimer’s disease (AD), as an alternative platform to understand disease-associated pathways and mechanisms [4]. Regarding age-related disorders, another outstanding review discussed the emerging field of NADomics, the high-throughput study of nicotinamide adenine dinucleotide (NAD+) and its related metabolites. As the NADome (NAD+ metabolome) represents an important biomarker for aging and neurodegenerative diseases, the authors suggested that NADomics can be used to elucidate the pathobiology of these disorders and identify potential therapeutic strategies [5]. Moreover, Perrone and collaborators aimed to review the most recent advancements in genomics, metabolomics, and proteomics related to sudden infant death syndrome (SIDS), which is characterized by an unexpected death during the sleeping period, typically occurring in infants under 1 year of age, and is associated with defects in the portion of the brain that controls breathing. These authors suggested that a model integrating different data from biomarkers and omics analyses may represent a valuable tool to identify a risk profile of SIDS in newborns [6]. Finally, another review addressed the role of zinc and its related proteins as important modulators of the epigenome in different chronic diseases, discussing their interaction with the chromatin [7].

Concerning neuropsychiatric disorders, an integrative multi-omics analysis identified four categories of key genes involved in the pathogenesis of schizophrenia (SCZ), thus offering new insights to better understand the complexity and regional heterogeneity of SCZ [8]. Moreover, another study suggested that the disruption of DGCR8-dependent microRNA biogenesis is crucial for the 22q11.21 copy number variant (CNV) genes involved in psychiatric disorders for late fetal cortical development [9]. MiRNomics has also been analyzed by some authors who described a panel of CSF-enriched miRNAs as a possible tool to identify and characterize new molecular signatures in different neurological diseases [10].

In regards to proteomics, Drastichova and collaborators performed a study in a rat brain after withdrawal from morphine, revealing that alterations in protein expression and phosphorylation are associated with synaptic plasticity and cytoskeleton organization, thus contributing to long-term neuroadaptations induced by drug use and withdrawal [11]. Additionally, another study used a combined approach of mass spectrometry-based label-free quantitative proteomics (LFQ) and bioinformatics to investigate the protective effect of *Orthosiphon stamineus* leaf proteins (OSLPs) in SH-SY5Y cells induced by H_2_O_2_ insults that have been prevalently reported in different neurological disorders [12]. Finally, some authors demonstrated the utility of chloride adducts for the examination of human brain lipidomics, as no single platform can evaluate lipidomics as a whole [13].

In conclusion, brain diseases, such as neurodegenerative diseases and mental disordes, need integrative understanding that draws a more reliable hypothesis for pathology, which can be accomplished by an in-depth study of molecular information [14,15,16]. However, molecular profiling, which makes it possible to understand brain diseases, has been relatively insufficient [17,18,19,20]. This Special Issue can provide important information to help gain an in-depth understanding of the molecular aspects of diverse brain diseases. Furthermore, it is believed that multi-omics analysis should be used for brain diseases because multi-omics technology includes multiple molecular profiling, metadata, and big data processing with informatics and computer science, so it is possible to provide new macroscopic, as well as microscopic, insights for understanding brain diseases [21,22,23].

## Data Availability

Not applicable.
